# Vitamins and Human Health: 3rd Edition Editorial

**DOI:** 10.3390/nu18172868

**Published:** 2026-09-02

**Authors:** Mackenzie Guettler, Tyler Barker

**Affiliations:** 1 Sports Medicine Research Institute, College of Medicine, The Ohio State University Wexner Medical Center, Columbus, OH 43202, USA; mackenzieguettler@gmail.com; 2 Department of Orthopaedics, University of Utah, Salt Lake City, UT 84108, USA

Vitamins are essential micronutrients that regulate numerous physiological responses critical to human health: cellular metabolism, immune function, endocrine signaling, tissue repair, musculoskeletal performance, and more. Classically, vitamins were studied in the context of deficiency and disease. However, as our knowledge grows, the understood scope expands, positioning vitamins as dynamic modulators of disease risk, progression, treatment responsiveness, and recovery. This third edition of “Vitamins and Human Health” elaborates upon previous editions by showcasing an evolving body of work, spanning molecular biology, clinical medicine, endocrinology, metabolism, rehabilitation, public health, and sports performance. The contributions featured in this Special Issue highlight the increasingly diverse roles and progressive understanding of vitamins across health and disease.

Vitamin D

Vitamin D is a pleiotropic micronutrient traditionally associated with calcium and phosphate homeostasis, but now recognized for its broader influence across systems. Reflecting the diverse physiological actions of vitamin D, nine articles included in this Special Issue examine its role across cardiovascular, respiratory, gastrointestinal, musculoskeletal, metabolic, endocrine, and developmental health.

Cardiovascular disease remains a leading cause of morbidity and mortality worldwide, promoting continued interest in identifying modifiable risk factors. Varzideh and colleagues [contribution 1] conducted a comprehensive review of vitamin D’s function in relation to cardiovascular disease and as a potential modifier for disease risk, integrating molecular, epidemiological, and clinical evidence. The collective body of evidence demonstrated that low circulating 25-hydroxyvitamin D concentrations are consistently associated with hypertension, atherosclerosis, myocardial infarction, heart failure, stroke, and cardiovascular mortality. Mechanistic investigations suggested that vitamin D influences endothelial function, oxidative stress, inflammatory signaling, and renin–angiotensin–aldosterone system activity. The review also discussed how factors such as baseline vitamin D status, intervention timing, dosing strategies, genetic variability, and disease comorbidities may contribute to inconsistent findings observed in supplementation trials. While these mixed results warrant further research, this review by Varzideh and colleagues bolsters growing attention to targeted vitamin D interventions.

Expanding the endocrine and metabolic actions of vitamin D, Paez-Allendes and colleagues [contribution 2] conducted a systematic review and meta-analysis of randomized controlled trials to gauge the effects of vitamin D supplementation on androgen-related outcomes in adult men. The authors surveyed evidence from 27 studies, including 21 comparison-level datasets, and assessed outcomes including total testosterone, sex hormone-binding globulin, free androgen index, calculated free testosterone, and bioactive testosterone. Despite observational evidence linking vitamin D status to androgen physiology, the meta-analysis showed no statistically significant effect of vitamin D supplementation on total testosterone or androgen bioavailability markers. Although vitamin D receptors are expressed throughout the male reproductive tract, supplementation alone may not meaningfully alter androgen status in adult men. Future high-quality randomized trials hold promise in clarifying the relationship between vitamin D and male reproductive endocrinology, with controlled supplementation protocols and demographic characteristics.

The immunomodulatory properties of vitamin D have also generated considerable interest in respiratory disease. An umbrella review by Liu and colleagues [contribution 3] evaluated systematic reviews and meta-analyses around vitamin D supplementation in children with asthma. The review concluded that vitamin D supplementation in children with asthma could improve asthma-related outcomes, reinforcing a role for vitamin D in pediatric respiratory care. Biological plausibility for this verdict comes from the established involvement of vitamin D in immune regulation and inflammatory responses. The variation in reviews and primary studies included by Liu et al. highlights the need for future high-quality randomized controlled trials. 

Săsăran and colleagues [contribution 4] extended the clinical relevance of vitamin D into pediatric neurology with two infant case reports, accompanied by a comprehensive review of the literature examining hypocalcemic seizures due to severe vitamin D deficiency. The study demonstrated that both infants (male, aged 4 months and 5 months) experienced profound vitamin D deficiency secondary to the complete absence of maternal and infant supplementation, resulting in hypocalcemia and seizure presentation. Treatment with calcium and vitamin D supplementation restored normal biochemical parameters and completely resolved symptoms. The accompanying review of 27 studies and case reports further bolsters maternal hypovitaminosis D as a common contributor to infant hypocalcemic seizures, stressing the importance of prenatal and early-life vitamin D prophylaxis for preventing potentially life-threatening neurological complications. As is common of case studies, the generalizability of evidence is limited by the study’s observational nature and small patient number, although the included literature review strengthens the clinical significance of these findings.

Expanding on vitamin D and chronic inflammatory disorders, Kafentzi and colleagues [contribution 5] explored vitamin D deficiency in inflammatory bowel disease patients at a tertiary referral center. The authors were able to identify a high prevalence of vitamin D deficiency in the inflammatory bowel disease cohort, as well as associations between low vitamin D status and disease-related clinical characteristics. The work by Kafentzi and colleagues buttresses vitamin D as a critical factor in immune-mediated disease, and again supports growing investigation into disease-specific targeted interventions. 

Moving from immune and inflammatory conditions, Bae and colleagues [contribution 6] showed that vitamin D could be associated with metabolic health during recovery from musculoskeletal injury. The researchers examined the relationship between serum 25-hydroxyvitamin D concentrations and metabolic syndrome in 219 adults following anterior cruciate ligament reconstruction. This retrospective study found that patients with metabolic syndrome exhibited lower serum vitamin D concentrations; as the number of metabolic syndrome components increased, the prevalence of vitamin D sufficiency decreased. Thus, vitamin D status could provide insight into cardiometabolic health following orthopedic injury with surgical reconstruction. While the study by Bae and colleagues supports the established association between vitamin D insufficiency and adverse metabolic profiles, causative conclusions should be interpreted with caution due to the retrospective design and potential residual confounding.

Vitamin D influences neuropsychological outcomes as well. Kowalcze and colleagues [contribution 7] evaluated sexual functioning and depressive symptoms in women receiving levothyroxine treatment for the hypothyroid phase of postpartum thyroiditis. Women with sufficient vitamin D status experienced greater improvements in sexual function and depressive symptoms than women with deficient or insufficient vitamin D. Moreover, the researchers saw an association between vitamin D sufficiency and favorable hormonal and clinical treatment responses, including thyroid antibody levels and selected endocrine parameters. Taken together, these findings suggest that vitamin D status may influence therapeutic responsiveness and quality-of-life outcomes in women with postpartum thyroid dysfunction. 

Additional evidence complementing the endocrine actions of vitamin D by Krysiak and colleagues [contribution 8] could point to vitamin D as an influencer of endocrine responses to metformin therapy. The researchers conducted a pilot study to determine whether vitamin D supplementation could modify the pituitary effects of metformin in postmenopausal women with subclinical hypothyroidism and normal vitamin D status, contributing to a growing body of literature supporting the biological actions of vitamin D beyond skeletal health. The findings suggest that vitamin D altered selected pituitary responses to metformin treatment, supporting an interaction between vitamin D and endocrine regulation in women with subclinical hypothyroidism. Larger studies could reinforce these findings. 

Extending the endocrine relevance of vitamin D, Kowalcze and colleagues [contribution 9] investigated whether maternal euthyroid autoimmune thyroiditis influences minipuberty in female offspring, and whether vitamin D and selenium supplementation during pregnancy may modify this effect. Infant females born to mothers with autoimmune thyroiditis were compared to controls; half of the mothers with thyroiditis received supplementation during pregnancy. Daughters of non-supplemented mothers exhibited lower luteinizing hormone, estradiol, and progesterone levels, with smaller uterine length and breast diameter, pointing to altered early reproductive axis development. These differences were not observed in the offspring of supplemented mothers, suggesting a potential protective role of maternal vitamin D and selenium supplementation. The findings of Kowalcze and colleagues expand the scope of vitamin D research into developmental endocrinology and maternal–fetal health, though larger prospective studies are necessary to validate these observations and elucidate the mechanisms involved.

Collectively, the remarkably multifaceted role of vitamin D is supported by the studies included in this Special Issue, contributing to growing bodies of research in cardiovascular, respiratory, gastrointestinal, metabolic, musculoskeletal, endocrine, and developmental health. Despite varying outcomes across study populations and designs, the findings of these papers consistently reinforce the significance of vitamin D status in both health and disease. Taken as a whole, these contributions strengthen preexisting bodies of literature and identify promising opportunities for future mechanistic, translational, and clinical research.

B Vitamins

B vitamins, a group of eight water-soluble micronutrients, are critical for energy metabolism, erythrocyte synthesis, cellular homeostasis, DNA synthesis and repair, and more. Due to their roles in cellular pathways, B vitamins contribute across body systems to the maintenance of cardiovascular, musculoskeletal, and neurological health and beyond. Emerging evidence continues to expand the role of B vitamins, as reflected in this Special Issue by the collection of studies examining relationships with sarcopenia, cardiovascular disease outcomes, and novel antioxidant mechanisms.

Vitamin B12 is critical to neurological health due to its essential role in maintaining myelin integrity and supporting neurotransmitter synthesis, with deficiencies often contributing to neurological dysfunction and broader clinical manifestations [1,2,3]. Baik [contribution 10] investigated prospective associations of serum vitamin B12, homocysteine, and ferritin concentrations with probable sarcopenia. Using prospective data from 1930 adults aged 45–76 years with normal muscle quantity at baseline, the investigators evaluated whether these biomarkers predicted future risk of muscle and strength loss. An association was found between alterations in vitamin B12-related metabolic markers and probable sarcopenia, contributing to previous understanding of nutritional status in musculoskeletal aging. Overall, Baik’s findings add to a growing interest and body of research in the identification of biomarkers relevant to functional decline in older adults. However, causal relationships cannot be established due to the observational nature of this study.

Ren and colleagues [contribution 11] delved into the influence of B vitamins on cardiovascular health, performing a systematic review and meta-analysis of 13 randomized trials from the past thirty years. Vitamins B6, B9, and B12 assist in homocysteine metabolism, making them a strategy of interest for reducing cardiovascular risk. This study compiled evidence from randomized trials investigating the effects of B-vitamin complex interventions on arterial thrombotic outcomes; the researchers reported inconsistent findings across studies. These mixed results posit the cardiovascular effects of B-vitamin supplementation as dependent upon population characteristics, baseline nutrition status, and outcome selection. Heterogeneity of results, as interpreted by Ren and colleagues, substantiates future studies aimed at identifying patient populations most likely to benefit from targeted B-vitamin interventions.

The role of B vitamins may extend beyond their established metabolic functions as well, impacting cellular defense mechanisms against oxidative stress. Kato and colleagues [contribution 12] reviewed evidence around the potential antioxidant role of vitamin B6 through pyridoxal-5′-phosphate-dependent metabolic pathways and subsequent activation of nuclear factor erythroid 2-related factor 2 (Nrf2) signaling. The authors describe mechanisms beyond the traditional co-enzymatic pathways that could potentiate a role for vitamin B6 and endogenous antioxidant systems, linking vitamin B6 metabolism to Nrf2-mediated cytoprotective pathways. Kato and colleagues effectively broaden the scope of vitamin B6 in physiological aging, sarcopenia, and chronic disease prevention with these compelling proposed mechanisms; however, additional clinical investigations are needed to better define the contribution of these pathways to human health. 

The studies included in this Special Issue contribute to the growing recognition of B vitamins’ influence on biological processes extending beyond classical deficiency states, and serve to elucidate the diverse physiological functions of B vitamins across musculoskeletal health, cardiovascular disease, and oxidative stress regulation. Promising developments in mechanistic understanding, influence, and proactive prevention continue to reveal new opportunities for understanding B vitamins in health maintenance and disease prevention, identifying gaps for future mechanistic, translational, and clinical investigation.

Vitamin K

Vitamin K is a fat-soluble nutrient widely recognized for its roles in coagulation, bone mineralization, and vascular health. Growing evidence has suggested that vitamin K could also influence glucose metabolism, insulin sensitivity, and cardiometabolic health. Research in this area was synthesized by Ahmed and colleagues [contribution 13], who conducted a meta-analysis of eight randomized controlled trials examining the effects of vitamin K supplementation on glycemic outcomes in individuals with prediabetes and type 2 diabetes mellitus. Results from these studies illustrated an association between vitamin K supplementation and significant reductions in fasting blood glucose, glycated hemoglobin (HbA1c), and HOMA-IR, with no significant effects observed for fasting insulin or β-cell function. Subgroup analyses suggested that metabolic effects varied according to vitamin K isoform, dose, and intervention duration; findings related to β-cell function were less consistent. More broadly, the findings translated by Ahmed and colleagues support a potential role for vitamin K in glycemic regulation and metabolic health, and provide a foundation for future investigation into the complexity of the physiological actions with larger, longer-term randomized investigations to define the therapeutic potential of vitamin K.

Emerging Roles of Other Vitamins

Additional vitamins, beyond vitamins D and K, continue to reveal biological functions extending well beyond their traditionally understood roles in preventing deficiency. The studies included in this section illustrate the use of vitamins as biomarkers of physiological stress, influencers of tissue repair, and windows into clinical vulnerabilities across diverse disease states. 

Yoon and colleagues [contribution 14] conducted a comprehensive narrative review examining vitamin C as a stress-responsive micronutrient with roles extending across tissue repair, immune regulation, vascular biology, oxidative stress, and clinical recovery. While much of the existing literature focuses on preventing deficiency, the review synthesized mechanistic and clinical evidence supporting vitamin C’s involvement in collagen biosynthesis, endothelial function, neurotransmitter metabolism, wound healing, orthopedic recovery, fatigue, cancer-supportive care, and dermatologic health, while distinguishing established physiological functions from areas where evidence remains preliminary. The review also discusses practical considerations around formulation, route of administration, dosing, and safety, advocating for individualized supplementation based on patient vulnerability rather than routine high-dose administration in vitamin C-replete individuals. 

Growing understanding of vitamin C reflects the appreciation of vitamins as active regulators in tissue repair and recovery; recent research delves further, suggesting that vitamins, such as retinol, could provide valuable prognostic information in clinical settings, and, in the case of retinol, serve as useful biomarkers of organ dysfunction and perioperative risk. A prospective single-center exploratory study by Olasińska-Wiśniewska and colleagues [contribution 15] explored whether perioperative vitamin levels differed in patients who developed cardiac surgery-associated acute kidney injury. Interestingly, patients who developed acute kidney injury exhibited significantly higher preoperative retinol concentrations, while vitamin E and 25-hydroxyvitamin D3 levels did not differ significantly between groups. Retinol concentrations were also positively correlated with perioperative creatinine levels and inversely with estimated glomerular filtration rate. Collectively, these findings suggest that elevated retinol could serve as a potential biomarker of renal vulnerability in cardiac surgery patients. Although confirmation in larger prospective cohorts is warranted, these findings introduce retinol as a promising biomarker and expand the discussion of fat-soluble vitamins into surgical risk stratification. 

Together, these investigations illustrate that vitamins can function not only as therapeutic targets, but also as biomarkers of physiological stress and clinical vulnerability. Extending this perspective from individual disease states to broader populations, the remaining studies examine how vitamin status, dietary patterns, and nutritional adequacy influence health across diverse populations and stages of life.

Population Health, Diet, and Performance

Vitamins are crucial regulators and therapeutic targets of disease and disorder, as demonstrated by many of the articles in this Special Issue. However, the body of research is changing to encompass broader systemic questions around healthy aging, nutritional adequacy, physical performance, and disease prevention. The studies included in the following section examine vitamin status across diverse populations and life stages, delving into the influence of vitamins on long-term health outcomes.

Numerous physiological changes encapsulate the aging process, altering nutrient status and susceptibility to chronic diseases. Zhao and colleagues [contribution 16] examined associations between vitamin status and age-related diseases in 2646 adult residents of the Pearl River Delta region in Guangdong, China. Researchers identified associations between vitamin A, B, E, B1, B2, B3, B5, B6, and B9 concentrations and cerebrovascular disease, coronary heart disease, hypertension, dyslipidemia, and type 2 diabetes mellitus. Zhao and colleagues demonstrated nonlinear relationships between several vitamin biomarkers and health outcomes. The complexity of the results indicates that optimal vitamin status is likely more complicated than simply preventing disease, highlighting the relevance of baseline biomarkers and physiological conditions for a bespoke approach to supplementation. Although the cross-sectional design of this study limits the establishment of causal relationships, the findings clearly establish the necessity for further investigation in this field. 

Arjol and colleagues [contribution 17] evaluated whether habitual dietary vitamin consumption was associated with vascular health among individuals with Long COVID, expanding population-level investigations of vitamin intake. In this cross-sectional study of 304 Long COVID patients, dietary vitamin intake was assessed using a validated seven-day dietary record and compared with measures of aortic stiffness and mixed central–peripheral arterial stiffness. Although vitamins B1 and B6 demonstrated nominal inverse associations with aortic stiffness, these relationships were no longer statistically significant after correction for multiple comparisons, and no meaningful associations were identified for mixed central–peripheral arterial stiffness. These findings highlight the complexity of nutritional influences on vascular health in patients with persistent post-COVID-19 symptoms and underscore the need for longitudinal investigations that incorporate biochemical vitamin status alongside dietary intake. 

Dietary patterns are one of the most important determinants of vitamin intake and nutritional adequacy. In a cross-sectional study, Petisco-Rodriguez and colleagues [contribution 18] examined the relationship between adherence to the Mediterranean diet, vitamin intake adequacy, body composition, and physical activity in Spanish university students. Greater adherence to the Mediterranean diet was associated with improved intake adequacy of vitamin C, retinol equivalents, carotenoids, and fiber, which in turn showed positive correlations with folate. High adherence to the diet was also inversely correlated with body weight and BMI. These findings reinforce the Mediterranean diet as an effective nutritional strategy for diet quality and healthy body composition in young adults.

Wiacek and colleagues [contribution 19] explored the role of vitamins in physical performance and athletic health in a narrative review of research findings from the past decade, summarizing relevant literature on the influence of vitamin supplementation (focusing on vitamins A, C, D, E, and the B-complex vitamins) for athletic performance, recovery, and overall health in physically active individuals. The authors were able to highlight specific circumstances in which targeted supplementation could be beneficial, including vitamin D deficiency in indoor athletes, vitamins C and E to attenuate exercise-induced muscle damage and oxidative stress, vitamin A for immune modulation, metabolic regulation, and mitochondrial function, and B-complex vitamins to support energy metabolism and red blood cell synthesis. Wiacek and colleagues stress that routine supplementation of baseline normal vitamin levels may not consistently improve performance outcomes, and that dietary optimization is the primary strategy for improved performance. Taken as a whole, the findings of Wiacek et al. reinforce the importance of an individualized, tailored approach to nutritional supplementation, and further high-quality, long-term, sport-specific research with standardized supplementation protocols.

A Mendelian randomization study by Shi and colleagues [contribution 20] sought to elucidate the potential influence of micronutrients on autoimmune disease by examining associations between type 1 diabetes risk and 17 micronutrients using genetic instrumental variables. Leveraging large-scale genome-wide association study datasets spanning multiple ancestral populations, the investigators applied a two-sample Mendelian randomization approach to evaluate potential causal relationships, while minimizing confounding and reverse causation inherent to traditional observation studies. The authors identified a significant positive association between genetically predicted potassium concentrations and increased type 1 diabetes risk, where zinc, vitamin B12, retinol, and α-tocopherol demonstrated only nominal associations; the remaining vitamins and minerals showed no evidence of substantial causal effects. Although the findings of Shi and colleagues require further confirmation, they contribute to growing research efforts to distinguish micronutrients with potential causal roles in autoimmune disease development. 

These studies illuminate the importance of vitamin status and nutritional adequacy across diverse populations and stages of life. The findings reinforce the broad relevance to human health, whether through healthy aging, athletic performance, or disease susceptibility, while identifying critical gaps for future population-based, preventative research.

Collectively, the studies included in this third edition of “Vitamins and Human Health” reinforce the increasingly multifaceted role of vitamins in human physiology and disease. The contributions presented in this Special Issue demonstrate the evolution of vitamins’ influence beyond their classical roles, with biological influence spanning cardiovascular, immune, endocrine, metabolic, musculoskeletal, and neurological health. While vitamin deficiency and insufficiency remain clinically important concerns, emerging evidence continues to broaden the focus toward understanding how vitamin status modulates disease risk, progression, treatment responsiveness, recovery, and physical performance. As the field continues to evolve, future research integrating mechanistic biology, longitudinal clinical outcomes, and personalized nutritional approaches will be essential for leveraging vitamins to optimize health and mitigate disease across populations. 

## References

[1] Umekar M., Premchandani T., Tatode A., Qutub M., Raut N., Taksande J., Hussain U.M. (2025). Vitamin B12 Deficiency and Cognitive Impairment: A Comprehensive Review of Neurological Impact. Brain Disord..

[2] Leishear K., Boudreau R., Studenski S., Ferrucci L., Rosano C., de Rekeneire N., Houston D., Kritchevsky S., Schwartz A., Vinik A. (2012). Vitamin B12 and Neurological Function: Is There a Threshold Level?. Neurology.

[3] Ali A.A.H., Mohamed F.H.A., Hago S., Elgali I.F.E., Mohammed H.E.S., Mirghani R.G. (2025). The Neurological Sequelae of Vitamin B12 Deficiency: A Systematic Review and Randomized Controlled Trial. Cureus.

